# Identification of PPAR‐related differentially expressed genes liver hepatocellular carcinoma and construction of a prognostic model based on data analysis and molecular docking

**DOI:** 10.1111/jcmm.18304

**Published:** 2024-04-23

**Authors:** Yumeng Wang, Shuqiang Li, Zihang Liu, Xuanzheng Li, Yifan Yu, Hao Liu

**Affiliations:** ^1^ Department of Organ Transplantation and Hepatobiliary The First Hospital of China Medical University Shenyang Liaoning China; ^2^ Department of General Surgery Shengjing Hospital of China Medical University Shenyang Liaoning China

**Keywords:** hepatocellular carcinoma, immunotherapeutic targets, liver hepatocellular carcinoma, peroxisome, prognostic model, peroxisome proliferator‐activated receptors, tumour mutational burden

## Abstract

Liver hepatocellular carcinoma (LIHC) is a significant global health issue with limited treatment options. In this study, single‐cell RNA sequencing (scRNA‐seq) data were used to explore the molecular mechanisms of LIHC development and identify potential targets for therapy. The expression of peroxisome proliferator‐activated receptors (PPAR)‐related genes was analysed in LIHC samples, and primary cell populations, including natural killer cells, T cells, B cells, myeloid cells, endothelial cells, fibroblasts and hepatocytes, were identified. Analysis of the differentially expressed genes (DEGs) between normal and tumour tissues revealed significant changes in gene expression in various cell populations. PPAR activity was evaluated using the ‘AUCell’ R software, which indicated higher scores in the normal versus the malignant hepatocytes. Furthermore, the DEGs showed significant enrichment of pathways related to lipid and glucose metabolism, cell development, differentiation and inflammation. A prognostic model was then constructed using 8 PPARs‐related genes, including FABP5, LPL, ACAA1, PPARD, FABP4, PLIN1, HMGCS2 and CYP7A1, identified using least absolute shrinkage and selection operator‐Cox regression analysis, and validated in the TCGA‐LIHC, ICGI‐LIRI and GSE14520 datasets. Patients with low‐risk scores had better prognosis in all cohorts. Based on the expression of the eight model genes, two clusters of patients were identified by ConsensusCluster analysis. We also predicted small‐molecule drugs targeting the model genes, and identified perfluorohexanesulfonic acid, triflumizole and perfluorononanoic acid as potential candidates. Finally, wound healing assay confirmed that PPARD can promote the migration of liver cancer cells. Overall, our study offers novel perspectives on the molecular mechanisms of LIHC and potential areas for therapeutic intervention, which may facilitate the development of more effective treatment regimens.

## 1 INTRODUCTION

Hepatocellular carcinoma (HCC) is a highly prevalent and aggressive primary liver malignancy with high morbidity and mortality rates worldwide.^1^ The incidence of HCC has risen globally in recent years. An estimated 906,000 new cases of primary liver cancer, of which 75%–85% were HCC, and 830,000 deaths were recorded in 2020 alone.^2^ HCC is primarily treated by surgical resection, interventional therapy, liver transplantation, radiation therapy and systemic therapy.^3^ Although patients in the early stage of HCC can be cured by hepatectomy and liver transplantation, their 5‐year survival rate is below 70%.^4^ In addition, most patients are diagnosed at an advanced stage, when therapeutic interventions are limited and mainly consist of systemic therapy. Sorafenib and regorafenib are currently the standard drugs used for treating patients with advanced HCC.^5^, ^6^ Therefore, it is crucial to develop novel drugs and explore alternative therapeutic strategies in order to enhance the overall survival (OS) and quality of life of HCC patients.

Peroxisome proliferator‐activated receptors (PPARs) are a family of ligand‐activated transcription factors that comprise of three homologous members, PPARα, PPARβ/δ and PPARγ.^7^ PPARs regulate various metabolic pathways in distinct tissues.^8^ For instance, PPARα mainly participates in lipid metabolism, especially the transport, lipidation and oxidation of fatty acids in the liver.^9^ PPARβ/δ is mainly associated with fatty acid oxidation and maintenance of blood cholesterol and glucose levels,^10^ while PPARγ regulates adipose cell differentiation, lipid storage and insulin sensitivity.^11^, ^12^ As transcription factors, PPARs regulate the expression of various target genes, especially those linked to lipid/glucose metabolism, inflammation and cell differentiation.^13^, ^14^, ^15^ PPARs can be activated by corresponding PPAR ligands, and activated‐PPAR combine with retinoid X receptors (RXR) to form a heterodimer, which further recognizes distinct DNA sequences in the promoter regions of the target genes.^16^, ^17^

Not surprisingly, dysregulation of PPARs has been linked to metabolic disorders and cancers, and the aberrant expression of PPARs is associated with tumour growth, angiogenesis, metastasis and immunity.^18^, ^19^ Currently, only a few studies have reported the role of PPAR in liver cancer. Chen et al. showed that 4‐phenylbutyric acid promotes HCC progression by activating PPAR and maintaining the cancer stem cells.^20^ Therefore, elucidating the role of PPARs in cancer progression can identify novel therapeutic targets. In this study, we established a robust prognostic model for HCC using PPARs‐related genes in order to predict the OS and identify promising therapeutic targets.

## 2 MATERIALS AND METHODS

### 2.1 Data retrieval

RNA microarray datasets (GSE14520, *n* = 177)^21^ and single‐cell RNA sequencing (scRNA‐seq) datasets (GSE149614, HCC samples = 10 and normal liver samples =8)^22^ were downloaded from Gene Expression Omnibus (GEO) database (https://www.ncbi.nlm.nih.gov/geo/). The Cancer Genome Atlas (TCGA)‐liver hepatocellular carcinoma (LIHC) (https://portal.gdc.cancer.gov/) dataset was downloaded from s‘TCGAbiolinks’ R package.^23^ Only patients with OS of more than 30 days were enrolled for the survival‐related analysis. The International Cancer Genome Consortium (ICGC)‐LIRI dataset (229 samples) was obtained from the ICGC database (https://icgc.org/) to validate the prognostic model. All ethical requirements for data accession and analysis were followed.

### 2.2 Analysis of scRNA‐seq data

The ‘Seurat’ R package^24^ was used for scRNA‐seq data analysis, normalization and visualization. The ‘Findmarkers’ function was used to screen for the differentially expressed genes (DEGs), with adjusted *p*‐value <0.05 and |log_2_fold‐change|>0.25 as the criteria. The R package ‘Dorothea’^25^ and ‘PROGENy’^26^ were used to determine transcription factor activity and pathway signatures associated with the expression of PPARs‐related genes. The PPAR scores of the cell clusters were calculated using R package ‘AUCell’, following publicly available code and tutorial (https://www.bioconductor.org/packages/devel/bioc/vignettes/AUCell/inst/doc/AUCell.html).

### 2.3 Analysis of bulk RNA‐seq data and mutation data

The ‘limma’ R package^27^ was used to identify DEGs based on adjusted *p*‐value <0.05 and |log_2_fold‐change|>1. The ‘maftools’ R package was used to visualize mutation data. The frequency of tumour‐infiltrating cells was calculated using the ‘IOBR’ R package.^28^

### 2.4 Construction of the PPARs‐related gene signature and unsupervised clustering

The prognostic PPARs‐related genes were screened by Least Absolute Shrinkage and Selection Operator (LASSO)‐Cox regression analysis using the ‘glmnet’ R package.^29^ The risk score for each patient was calculated based on the expression levels of eight model genes and their corresponding risk coefficients using the following equation:

$$\text{Risk score}=\Sigma \left(\mathrm{\beta i}\times \mathrm{Ei}\right)$$

*βi* signifies the hazard coefficient and *Ei* denotes the magnitude of the expression of the gene. With median risk score as the cutoff, the LIHC patients were classified into low‐ and high‐risk groups. Kaplan–Meier (K–M) survival analysis was performed using ‘survival’ and ‘survminer’ R packages. The ‘ConsensusClusterPlus’ R package was used to perform consensus clustering based on the model genes.

### 2.5 Pathways enrichment analysis

The ‘clusterProfiler’ R package was used for pathway enrichment analysis,^30^ with adjusted *p*‐value <0.05 as the threshold. The R package ‘GSVA’ was employed to perform gene set variation analysis (GSVA) on hallmark gene sets within each cell cluster.^31^

### 2.6 Drug prediction and molecular docking

The drug molecules targeting the model genes were identified using web porta Lod Enrichr (https://maayanlab.cloud/Enrichr/) and the Drug Signatures Database (DsigDB). The corresponding small molecular structure (mol format) was downloaded from the PubChem database according to the name of a small molecule. To optimize the stereo‐conformation of the small molecule structures, RDKit was used to invoke the MMFF94 force field, and the optimal conformation and energy were calculated. Protein structures were derived from AlphaFold2 or PDB structure databases (https://www.rcsb.org/), and the docking software Smina was used for molecular docking. The centers and fractions of docking proteins are shown in Table 1. The approximation of docking was 80, and each time, 80 conformations were generated.

**TABLE 1:** Molecular docking parameters and fractions.

Drug	Gene	X‐center	Y‐center	Z‐center	Score (kcal/mol)
Perfluorohexanesulfonic acid	HMGCS2	0.713	3.246	0.000	−8.6
Perfluorohexanesulfonic acid	CYP7A1	−1.713	−3.246	−5.885	−7.5
Perfluorohexanesulfonic acid	PPARD	0.195	3.145	12.917	−7.2
Triflumizole	FABP4	0.194	3.142	2.443	−8
Triflumizole	LPL	5.832	−1.378	−7.614	−7.7
Perfluorononanoic acid	HMGCS2	0.713	3.246	0.000	−8.2
Perfluorononanoic acid	CYP7A1	−1.713	−3.246	−5.885	−7.1
Perfluorononanoic acid	PPARD	0.195	3.145	12.917	−7.2

### 2.7 Cell culture

The human non‐small cell lung cancer cell lines HepG2 and HUH7 were purchased from the Cell Bank of the Chinese Academy of Sciences. HepG2 cells were cultured in minimum essential medium (MEM; Invitrogen; Thermo Fisher Scientific) containing 10% fetal bovine serum (FBS), while HUH7 cells were cultured in Dulbecco's modified Eagle's medium (DMEM; Invitrogen; Thermo Fisher Scientific) containing 10% FBS. The cells were incubated at 37°C in a 5% CO_2_ environment.

### 2.8 Transfection

Two siRNAs specific for PPARδ were designed and synthesized by GIMA Corporation. The cells were seeded in a six‐well plate at the density of 1 × 10^5^ cells/well in 2 mL medium, and incubated for 24 h. A mixture of 1.3 μg siRNA and 4.6 μL transfection reagent (PolyFast, HY‐K1014; MCE) was prepared and allowed to stand at room temperature for 15 min. The mixture was then added to the corresponding wells, which were shaken thoroughly to ensure uniform mixing. The medium was replaced 6 h later, and the cells were cultured for 48 h. The siRNA sequences were as follows:

NC sense: 5′‐UCCUCCGAACGUGUCACGUTT‐3′;

NC anti‐sense: 5′‐ACGUGACACGUUCGGAGAATT‐3′;

Si1 sense: 5′‐ATTATTTCACCAGCAGCAT‐3′;

Si1 anti‐sense: 5′‐TATTCATTGCGGCCATCAT‐3′;

Si2 sense: 5′‐TCAAGAAGACCGAAACCGA‐3′;

Si2 anti‐sense: 5′‐GCAAACCCTTCAGTGATAT‐3′.

### 2.9 CCK‐8 assay

The transfected cells were seeded in a 96‐well plate at the density of 2 × 10^3^ cells. After 2‐, 24‐, 48‐, 72‐, and 96 h of culture, CCK8 reagent was added to each well in complete medium at the final volume of 200 μL. The cells were incubated for 1 h in the dark, and the absorbance of each well was measured at 450 nm using a spectrophotometer. The experiment was repeated thrice, and each group had three replicates.

### 2.10 Wound healing assay

The transfected cells were cultured till confluent, and the monolayer was scratched in the center of the well with a sterile 200 μL pipette tip. After washing twice with phosphate‐buffered saline to remove the dislodged cells, fresh serum‐free medium was added, and the cells were cultured for 48 h. Images of the scratched region were taken at 0 and 48 h, and the wound coverage was measured using ImageJ software.

### 2.11 Statistical analysis

Wilcoxon test or Kruskal–Wallis test was used to compare groups. All statistical analyses were performed using R software (4.2.2). *p* < 0.05 was considered statistically significant.

## 3 RESULTS

### 3.1 Single‐cell RNA profile of LIHC

**FIGURE 1:**
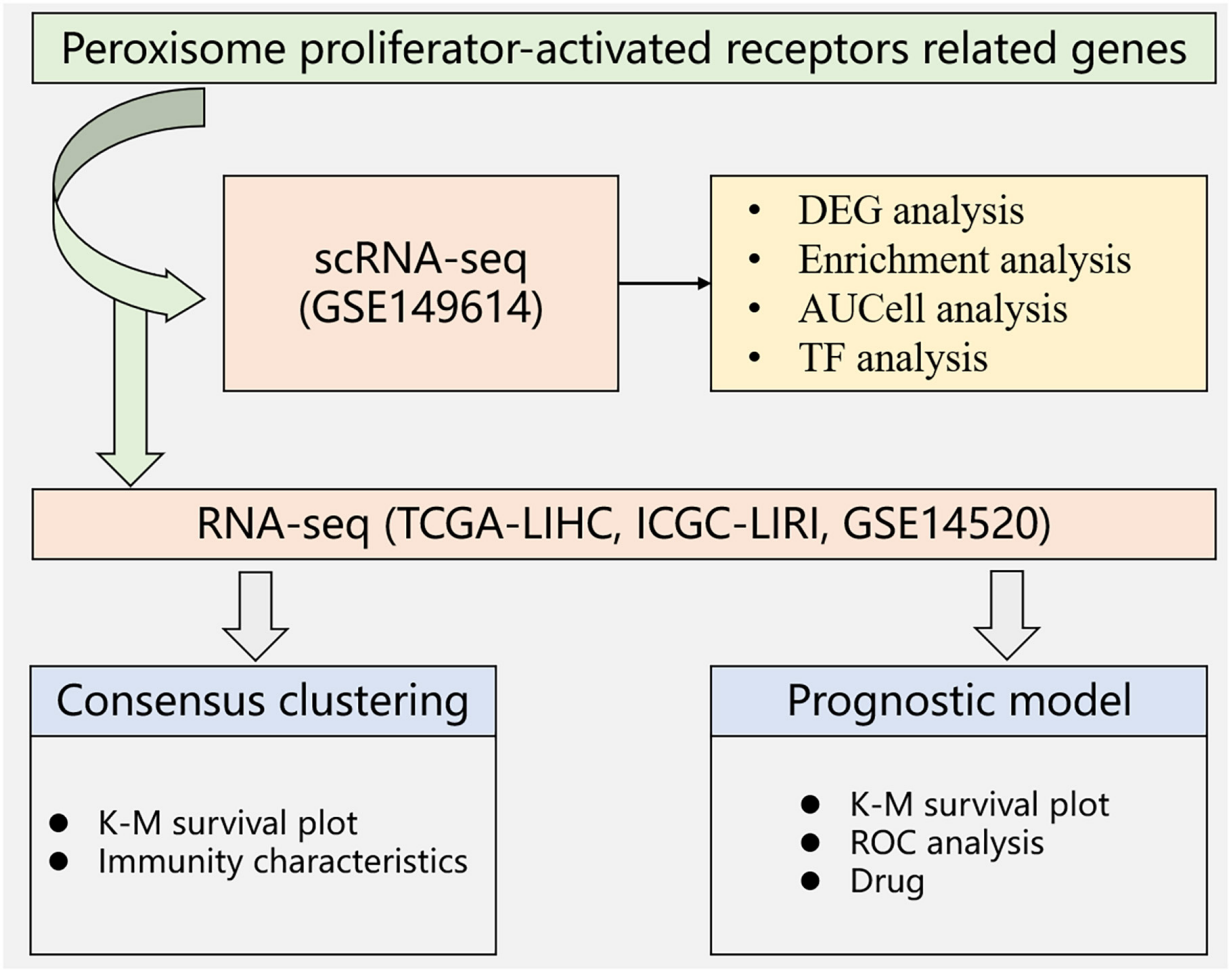
Flowchart of the overall workflow of this study.

**FIGURE 2:**
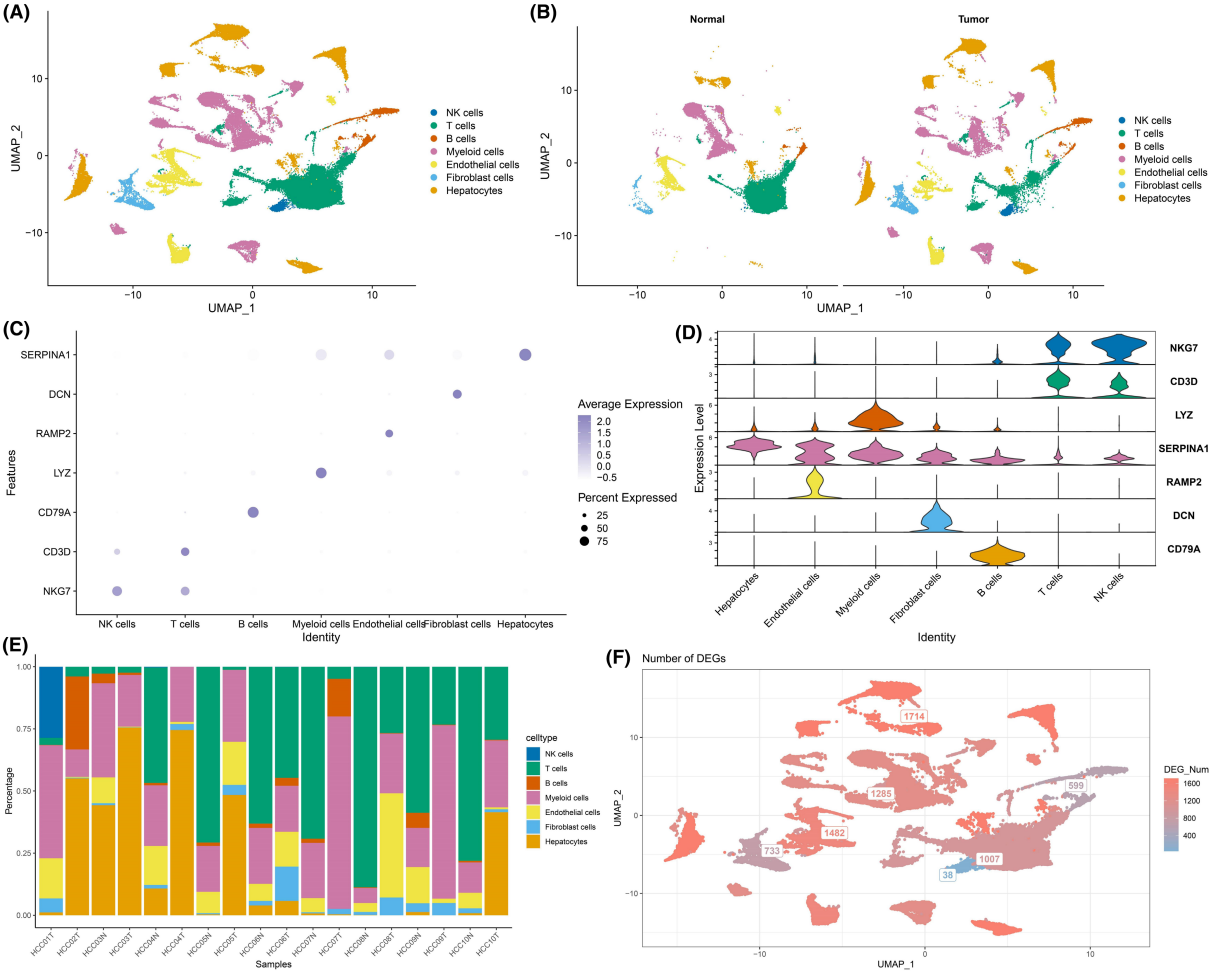
Analysis of scRNA‐seq data. (A) Seven cell clusters were identified with the Uniform Manifold Approximation and Projection (UMAP) dimensionality reduction algorithm. Each colour represents the annotated phenotype of each cluster. (B) UMAP plot of normal and tumour groups. (C) Dot plot of cell type marker genes. The colours of the dots represent the average expression, and the size of the dots represents the average percentage of gene expression. (D) Violin plot showing the distribution of cell type marker genes in each cell type. (E) Bar plot showing cell type distribution in each sample. (F) UMAP plot showing the number of DEGs in each cell type (tumour vs. normal).

The workflow of the study is shown in Figure 1. We identified clusters of NK cells, T cells, B cells, myeloid cells, endothelial cells, fibroblasts and hepatocytes in LIHC samples based on the scRNA‐seq data of GSE149614 (Figure 2A). The distribution of these populations in tumour tissues and normal tissues are shown in Figure 2B, and their proportion in each LIHC sample is shown in Figure 2E. The marker genes of each cell population are presented in Figure 2C,D. We also screened for the DEGs between tumour and normal samples in the distinct cell groups, and detected the highest number of DEGs in the hepatocytes (Figure 2F). The nFeature_RNA (number of genes per cell) exhibited a positive correlation with nCount_RNA (UMI per cell), with a correlation coefficient of 0.91 (Figure S1A). The expression profiles of each sample are presented in Figure S1B. Principal component analysis showed no significant batch effect (Figure S1C).

### 3.2 Evaluation of the PPAR‐related gene score

**FIGURE 3:**
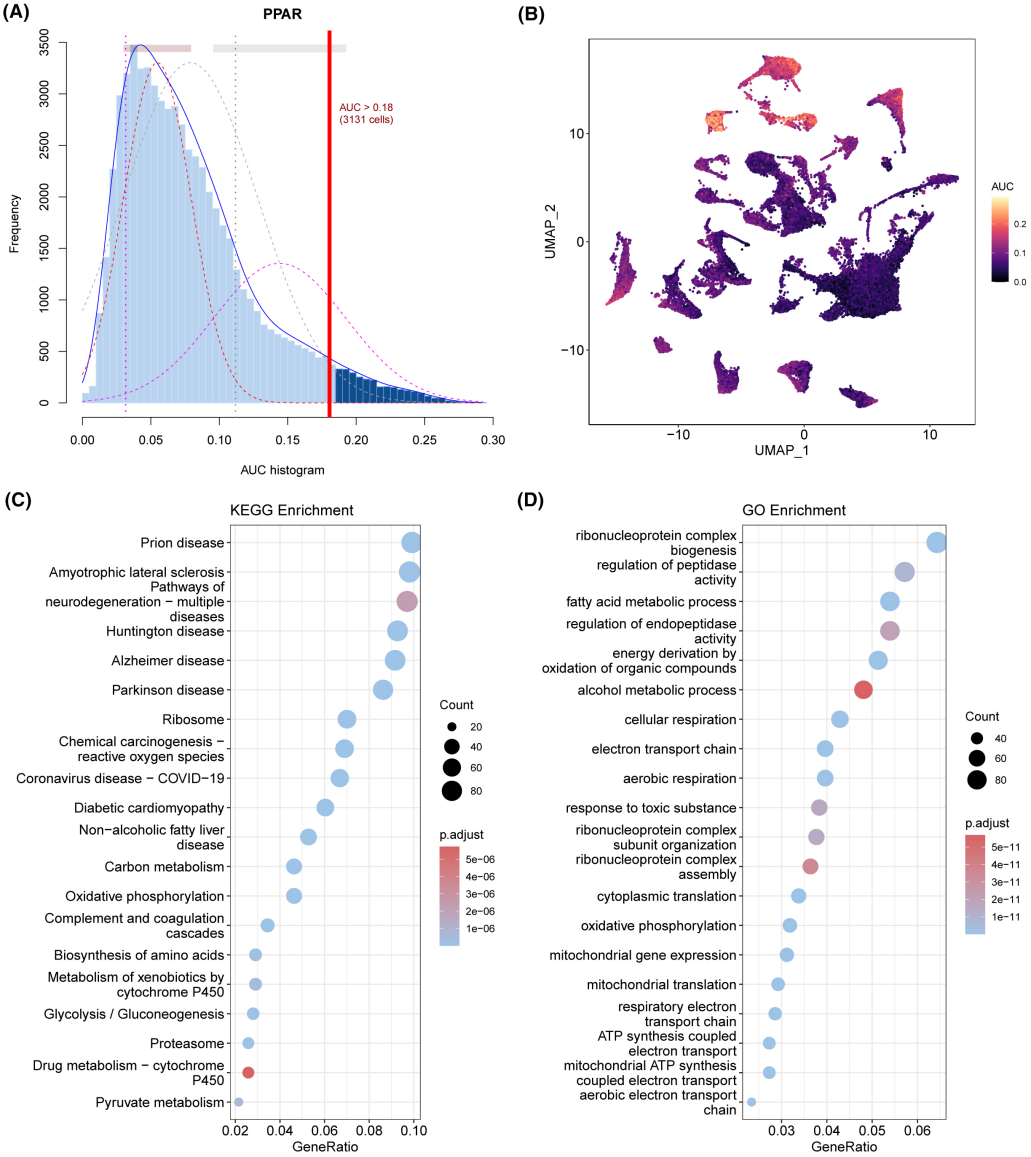
PPAR scores of cell types in liver hepatocellular carcinoma (LIHC). (A) The scores of PPAR‐related genes. The threshold was chosen as 0.18. (B) UMAP plots of PPAR scores in all cell types. Hepatocytes expressed more genes and exhibited higher area under the curve (AUC). (C) KEGG analysis of the DEGs in the hepatocytes. (D) Gene Ontology analysis of DEGs in the hepatocytes.

The activity of the PPARs in each cell population was analysed using the AUCell R software (Figure 3A). The area under the curve (AUC) was greater for the cells with higher gene expression. As shown in Figure 3B, the AUC value of hepatocytes was the highest, suggesting that the PPAR score of hepatocytes was higher compared to the other cell types. Therefore, we screened for the DEGs between the hepatocytes of the tumour and normal liver samples, and functionally annotated them by Gene Ontology (GO) and Kyoto Encyclopedia of Genes and Genomes (KEGG). As shown in Figure 3C,D, the DEGs were mainly related to lipid and glucose metabolism, cell development and differentiation, and inflammation. In addition, compared to normal hepatocytes, the PPAR score of tumour hepatocytes was lower (Figure S2).

### 3.3 Pathway enrichment analysis of the annotated genes in LIHC

**FIGURE 4:**
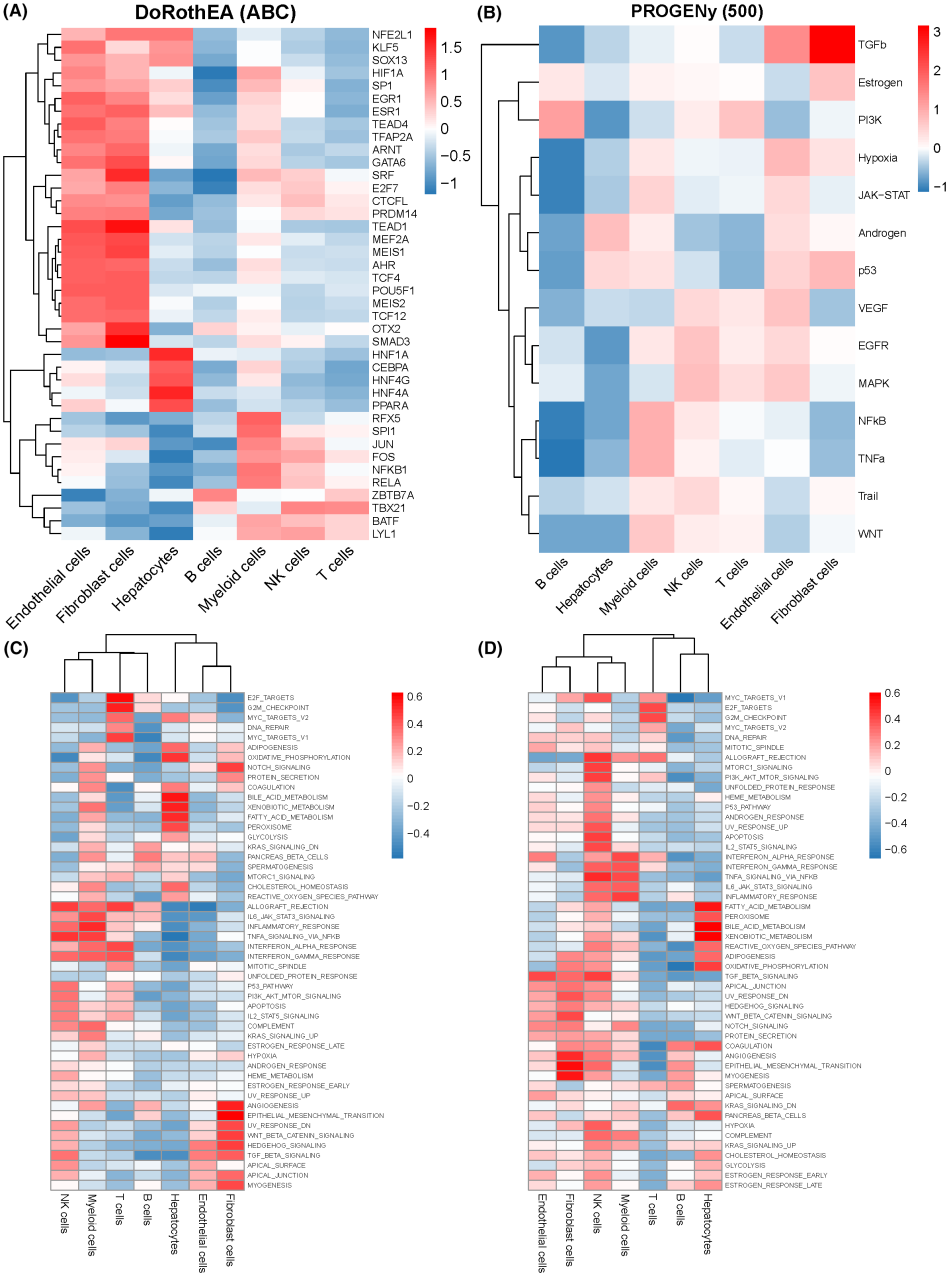
Transcription factor analysis and functional enrichment. (A) Heatmap showing the VIPER‐inferred protein activity for the top 40 regulatory proteins in each cell type. (B) Heatmap showing ProgenY scores of the tumour pathways in major liver hepatocellular carcinoma (LIHC) cell types. (C) The results of gene set variation analysis (GSVA) of hallmark pathways in the normal samples. (D) The results of GSVA of hallmark pathways in the tumour samples.

The top 30 differentially expressed transcription factors in each cell population are shown in Figure 4A. The correlation between the expression levels and tumour‐related pathways was analysed by PROGENy (Figure 4B), and the GSVA scores of 50 hallmark pathways were calculated for the different cells in normal and tumour tissues (Figure 4C). In the normal liver tissue, the enriched pathways included PANCREAS_BETA_CELLS, SPERMATOGENESIS, ALLOGRAFT_REJECTION, IL6_JAK_STAT3_SIGNALLING, and so on (Figure 4D). DNA REPAIR, ANDROGEN_RESPONSE, INTERFERON_ALPHA_RESPONSE, ADIPOGENSIS, TGF_BETA_SIGNALLING, COAGULATION and ANGIOGENESS were enriched in the HCC samples (Figure 4E). Furthermore, the top 10 transcription factors were highly expressed in normal tissues, while the remaining 20 transcription factors showed moderate expression in tumour tissue (Figure S3A). The normal tissues were enriched in nuclear factor‐kappa B, phosphoinositide 3‐kinase, and Trail pathways, while the androgen, JAK–STAT, and p53 signalling pathways were enriched in the tumours samples (Figure S3B).

### 3.4 Identification of PPAR‐related DEGs in the HCC patients

**FIGURE 5:**
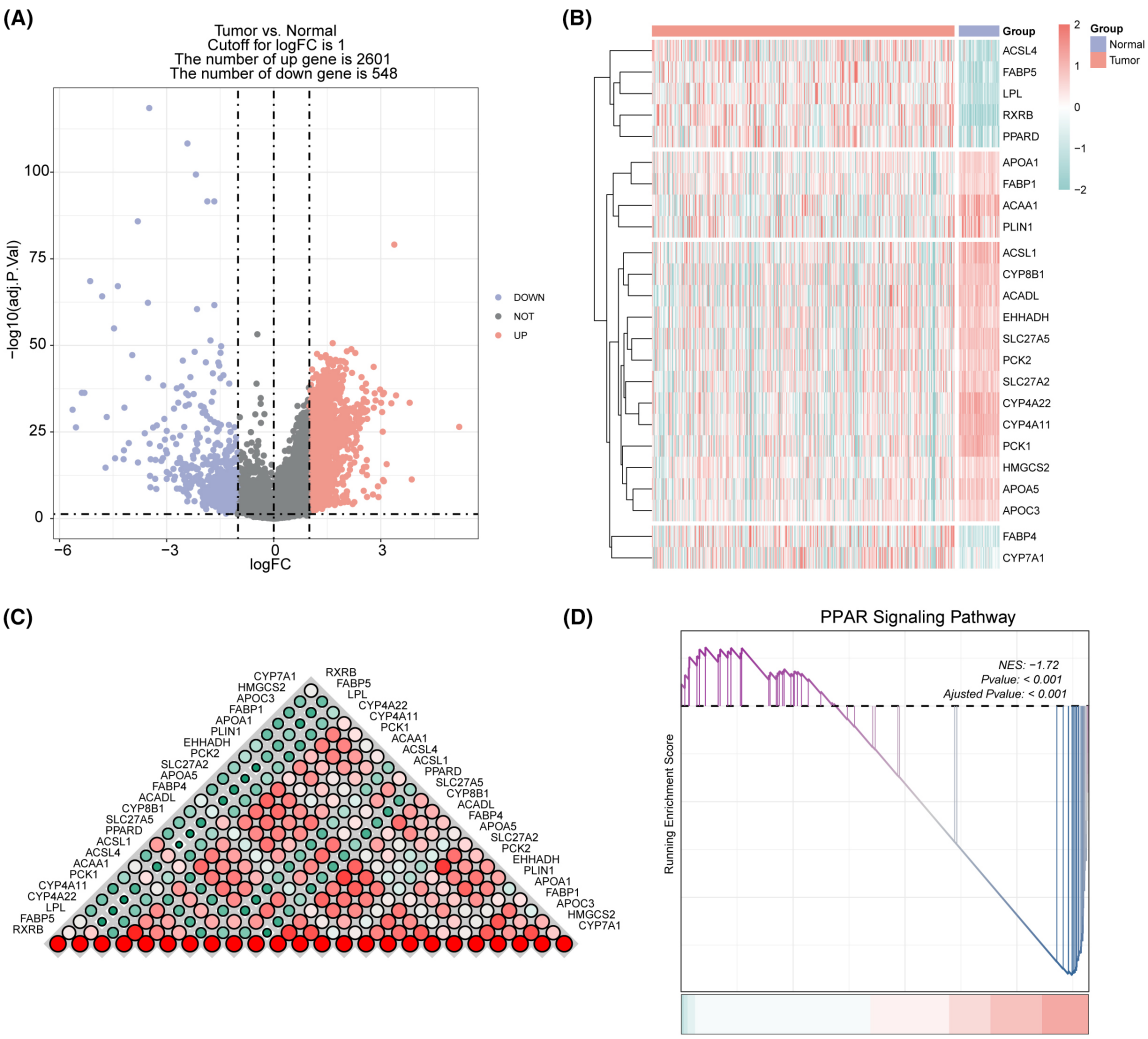
Transcriptional features in liver hepatocellular carcinoma (LIHC). (A) Volcano plot of DEGs between tumour and normal samples. Red dots represent up‐regulated genes and purple dots represent down‐regulated genes. (B) Heatmap of differentially expressed PPAR‐related genes in the tumour and normal samples. (C) The correlation among 24 differentially expressed PPAR‐related genes. (D) gene set enrichment analysis (GSEA) of PPAR signalling pathways in tumour and normal samples.

**FIGURE 6:**
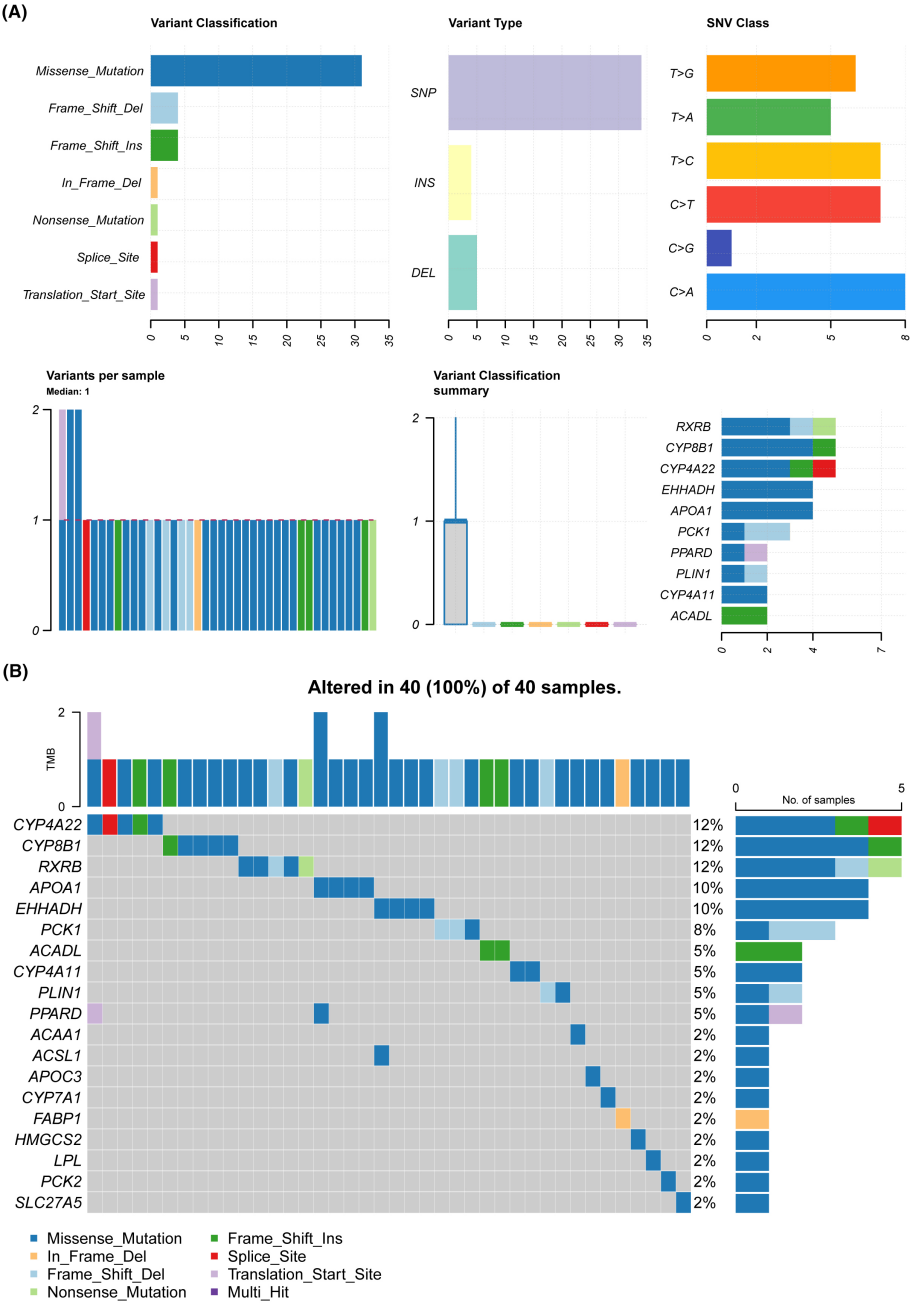
The top 20 frequently mutated PPAR‐related genes. (A, B) An oncoplot of PPAR‐related genes in The Cancer Genome Atlas cohort.

We identified 3149 DEGs in the TCGA‐LIHC cohort, of which 2601 were up‐regulated and 548 were down‐regulated (Figure 5A). In addition, 24 PPAR‐related genes were differentially expressed between normal and tumour tissues (Figure 5B). The correlation of each PPAR‐related DEG was analysed and visualized using the correlogram (Figure 5C). GSEA revealed significant down‐regulation of the PPAR signalling pathway in tumour tissues, which was consistent with scRNA‐seq analysis (Figure 5D). Furthermore, missense mutations, single nucleotide polymorphisms, and single nucleotide variants of C>A, C>T, T>C were the most prevalent mutations in the PPAR‐related DEGs (Figure 6A). The top 20 mutations of PPAR‐related DEGs and mutations in 100% (40/40) of LIHC samples are shown in Figure 6B. The top three mutated genes were CYP4A22, CYP8P1 and RXRB, with a mutation frequency of 12%, while that of the remaining genes ranged from 2% to 10%.

### 3.5 Construction of PPAR‐related prognostic model for LIHC

**FIGURE 7:**
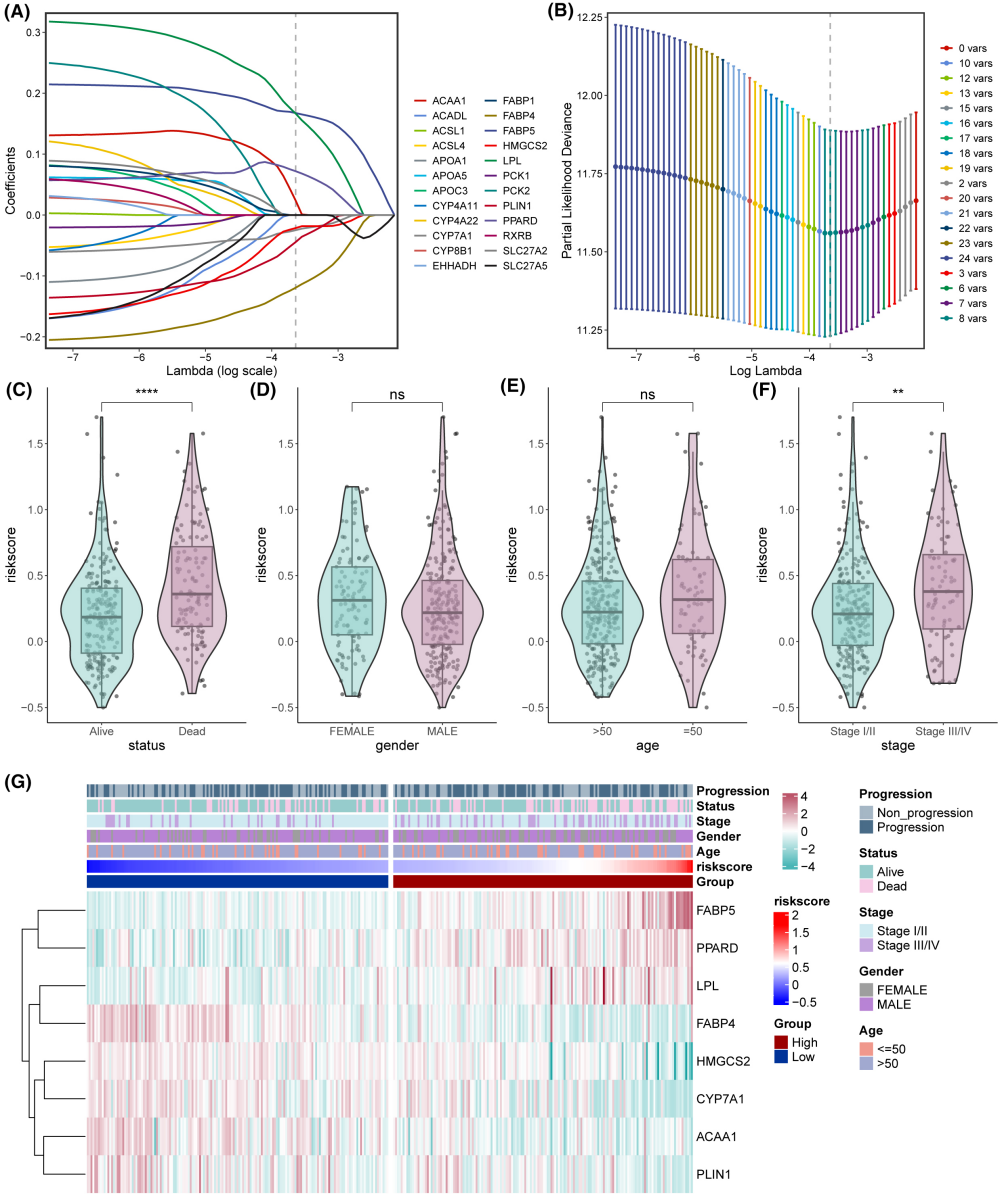
Construction of a prognostic gene signature for liver hepatocellular carcinoma (LIHC). (A) Selection of the eight model genes by machine learning method. (B) Cross‐validation of the constructed signature. (C–F) Violin plots of the relationship between risk‐score and survival status (C), gender (D), age (E), and clinical stage (F). (G) Heatmap of eight model genes and associated clinical features. *p < 0.05, **p < 0.01, ***p < 0.001, ****p < 0.0001, ns, not significant.

We screened eight PPAR‐related DEGs, including FABP5, LPL, ACAA1, PPARD, FABP4, PLIN1, HMGCS2 and CYP7A1, through LASSO‐Cox regression analysis to construct a prognostic model for LIHC (Figure 7A,B). The risk score was calculated as follows: 0.167448 × FABP5 + 0.166492 × LPL + 0.023651 × ACAA1 + 0.071884 × PPARD–0.11939 × FABP4–0.04441 × PLIN1–0.02141 × HMGCS2–0.02492 × CYP7A1. The prognostic model was associated with survival status and pathological stage (Figure 7C–F). Surviving patients had a lower risk score relative to the deceased patients (Figure 7C). Similarly, patients in the early stage of the disease also had lower risk scores than those with advanced disease (Figure 7F). Thus, HCC patients with a low‐risk score are likely to have a more favourable prognosis than those with high‐risk score. The differential expression of model genes and clinical characteristics in the low‐ and high‐risk patients are summarized in Figure 7G.

### 3.6 Evaluation and validation of prognostic model

**FIGURE 8:**
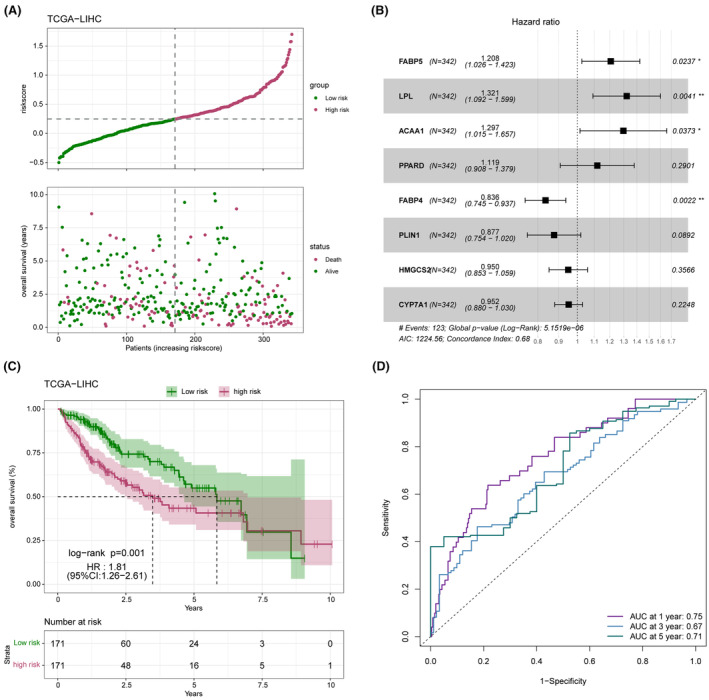
Performance of the PPARs‐related prognostic model in the TCGA‐liver hepatocellular carcinoma (LIHC) cohort. (A) High‐risk and low‐risk groups in the TCGA‐LIHC cohort. (B) Multivariate analysis for the eight model genes in the TCGA‐LIHC cohort. (C) Kaplan–Meier survival curves of the risk groups in TCGA‐LIHC cohort. (D) Receiver operator characteristic (ROC) analysis of risk‐score in TCGA‐LIHC cohort.

**FIGURE 9:**
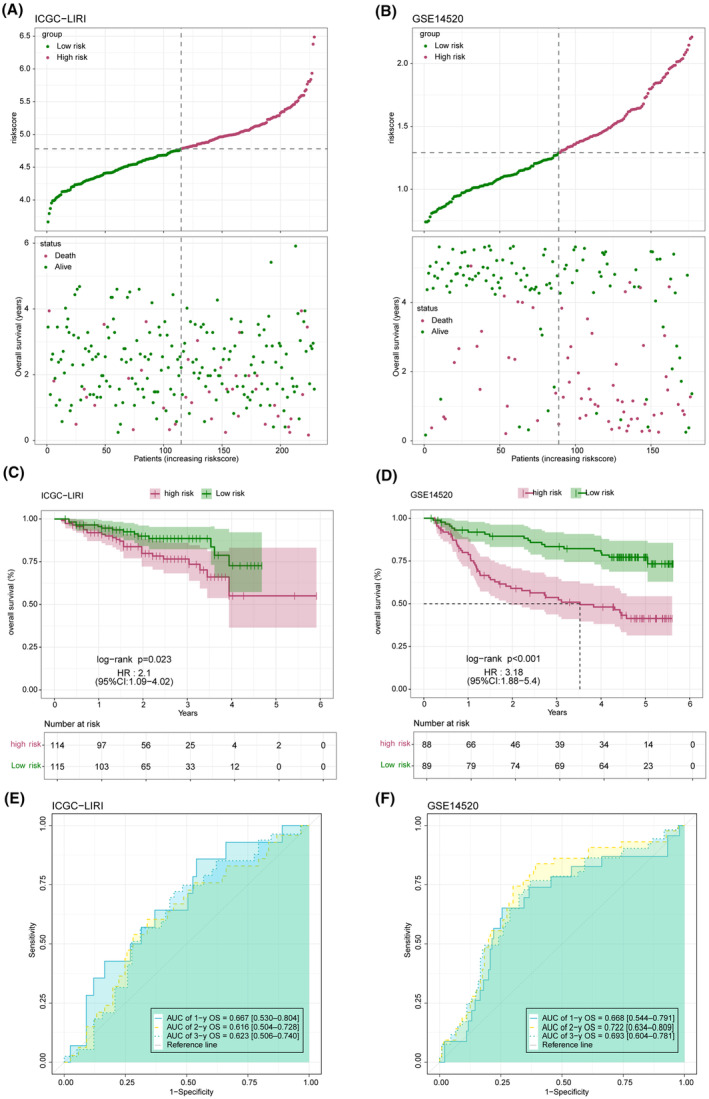
Validation of the prognostic model in ICGC‐LIRI and GSE14520 cohorts. (A, B) Comparison of the high‐ and low‐risk groups in the ICGC‐LIRI (A) and GSE14520 (B) cohorts. (C, D) Kaplan–Meier survival curves of the risk groups in the ICGC‐LIRI (C) and GSE14520 (D) cohorts. (E, F) Receiver operator characteristic (ROC) analysis of risk score in ICGC‐LIRI (E), and GSE14520 (F) cohorts.

The distribution of the risk score in the TCGA‐LICH dataset is shown in Figure 8A. Multivariate Cox regression analysis identified FABP5, LPL, ACAA1 and PPARD as risk factors, and FABP4, PLIN1, HMGCS2 and CYP7A1 as the protective factors of LIHC (Figure 8B). As shown in Figure 8C, the low‐risk group had a more favourable prognosis than the high‐risk group. In addition, receiver operating characteristic (ROC) curves showed that the prognostic model can predict 1‐, 2‐, and 3‐year survival of LIHC patients with high accuracy (Figure 8D). The validity and applicability of the prognostic model were further verified using the ICGI‐LIRI and GSE14520 datasets, and the risk‐score distribution in these datasets is shown in Figure 9A,B. Patients in the low‐risk group had significantly better prognosis in both ICGI‐LIRI and GSE14520 datasets (Figure 9C,D). Furthermore, the ROC curves of the prognostic model indicated high AUC values in the ICGI‐LIRI and GSE14520 datasets (Figure 9E,F). In summary, the prognostic model can accurately and reliably predict the prognosis of LIHC patients.

### 3.7 Consensus clustering and immune characteristics

**FIGURE 10:**
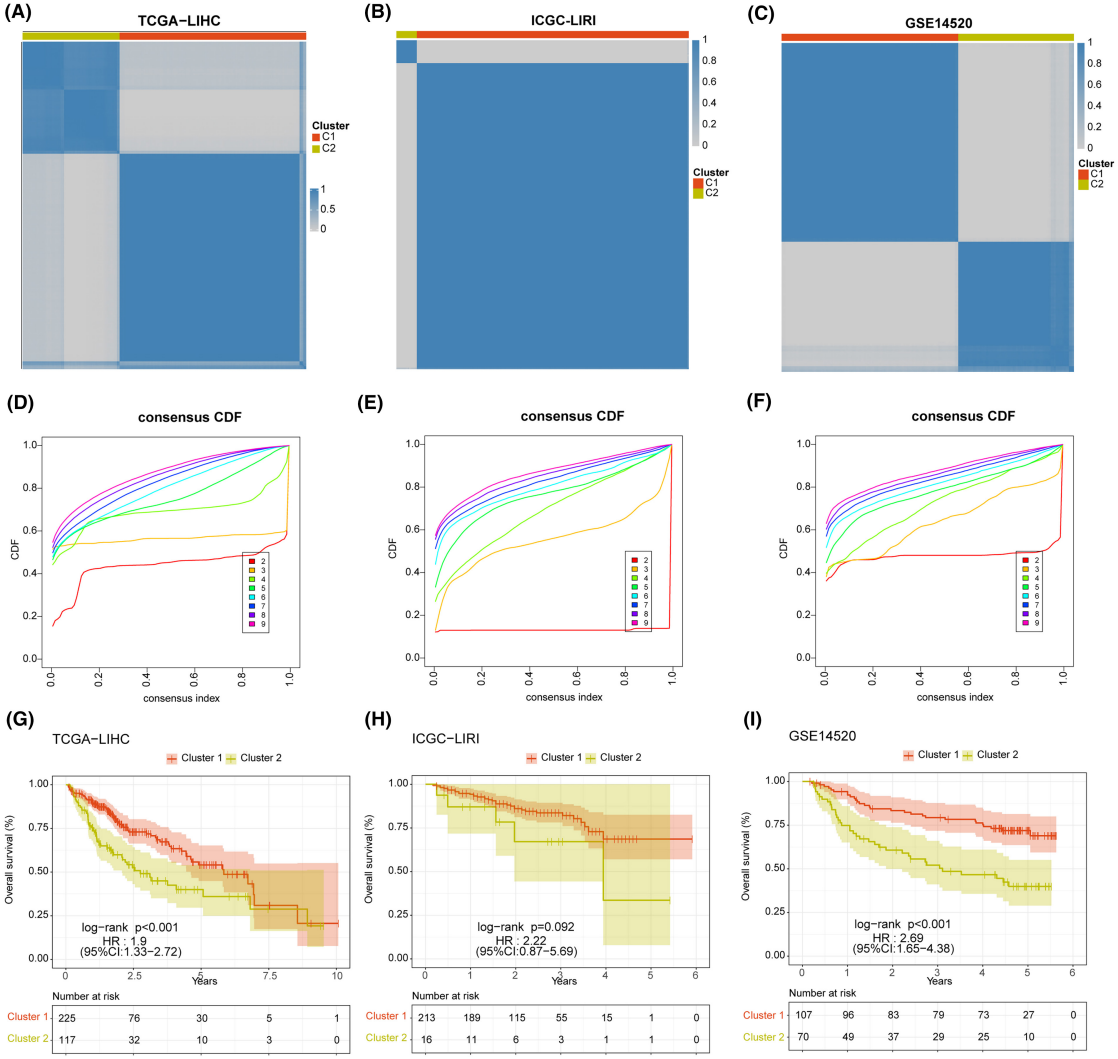
The results of consensus clustering based on model genes. (A–C) liver hepatocellular carcinoma (LIHC) patients of the TCGA‐LIHC (A), ICGC‐LIRI (B) and GSE14520 (C) cohorts were grouped into two molecular clusters when *k* = 2. (D–F) Empirical cumulative distribution function (CDF) plot displaying consensus distributions for each *k*‐value (from 2 to 9) in TCGA‐LIHC (D), ICGC‐LIRI I and GSE14520 (F) datasets. (G–I) Kaplan–Meier survival curves of patients in two different molecular clusters from TCGA‐LIHC (G), ICGC‐LIRI (H) and GSE14520 (I) cohorts.

**FIGURE 11:**
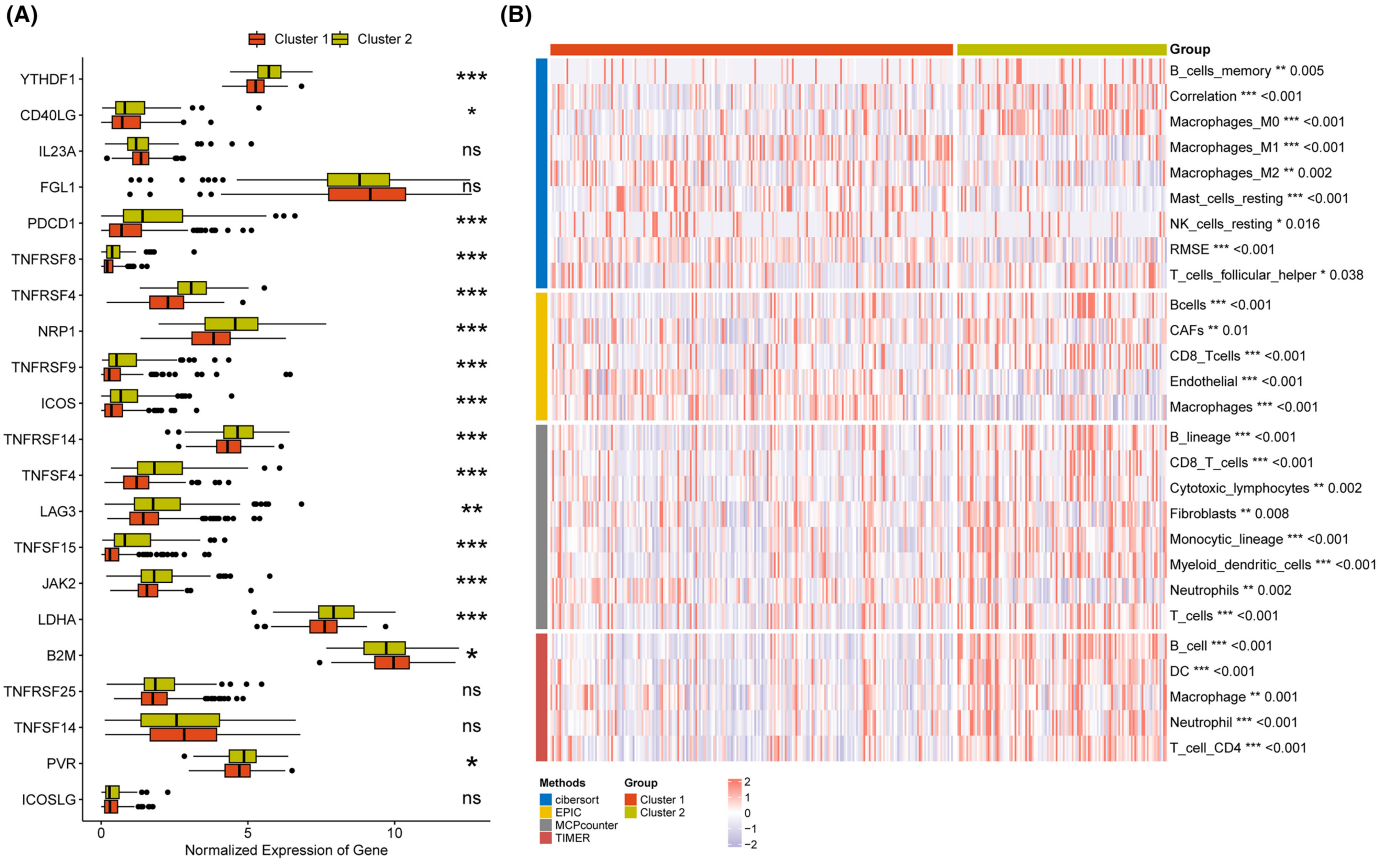
Tumour immune landscape of liver hepatocellular carcinoma (LIHC) patients from the two different molecular clusters. (A) Boxplot showing expression levels of immune checkpoint genes in Cluster 1 and Cluster 2. (B) Heatmap showing the distribution of infiltrating immune cells in LIHC patients from Cluster 1 and Cluster 2. *p < 0.05, ** *p* < 0.01, ***p < 0.001, ****p < 0.0001, ns, not significant.

**FIGURE 12:**
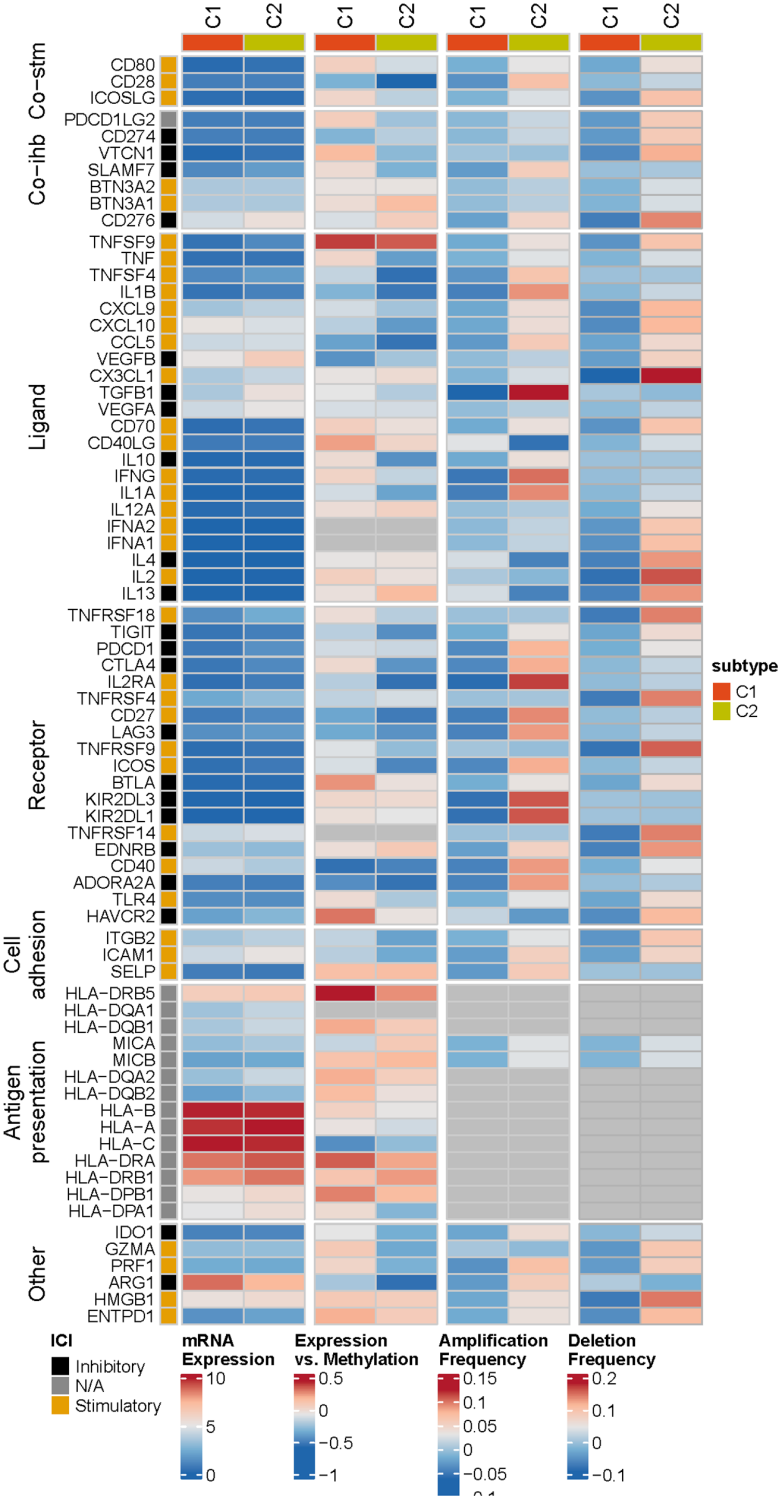
Regulation of immunomodulators in liver hepatocellular carcinoma (LIHC) patients from Cluster 1 and Cluster 2. Heatmap showing mRNA expression, expression versus methylation, amplification frequency, and the deletion frequency for immune‐related genes in the PPAR‐based clusters.

Based on the expression of eight model genes, patients in TCGA‐LIHC, ICGI‐LIRI, and GSE14520 datasets were classified into two clusters (C1 and C2) using the ‘ConsensusClusterPlus’ package (Figure 10A–C). The consensus cumulative distribution function (CDF) in the different clusters is shown in Figure 10D–F. Patients in Cluster 1 showed better survival outcomes compared to those in Cluster 2 in all datasets (Figure 10G–I). Furthermore, most immune checkpoint genes, including YTHDF1, PDCD1, TNFRSF4/8/14, NRP1, TNFSF4/15, LAG, JAK2, and so forth, were highly expressed in Cluster 2 (Figure 11A). The immune landscape in both clusters was analysed using the CIBERSORT, EPIC, MCPcounter and TIMER algorithms, which showed higher immune cell infiltration in Cluster 2, including that of M0 macrophages, B cells, CAFs, CD8 T cells, cytotoxic lymphocytes, myeloid dendritic cells, and so forth. (Figure 11B). As shown in Figure 12, there were significant differences in the expression levels, methylation, and amplification and deletion frequencies of immunomodulatory genes between Clusters 1 and 2, especially for genes encoding co‐stimulatory and co‐inhibitory molecules, ligands, and receptors.

### 3.8 Small molecule drug prediction of model genes

**FIGURE 13:**
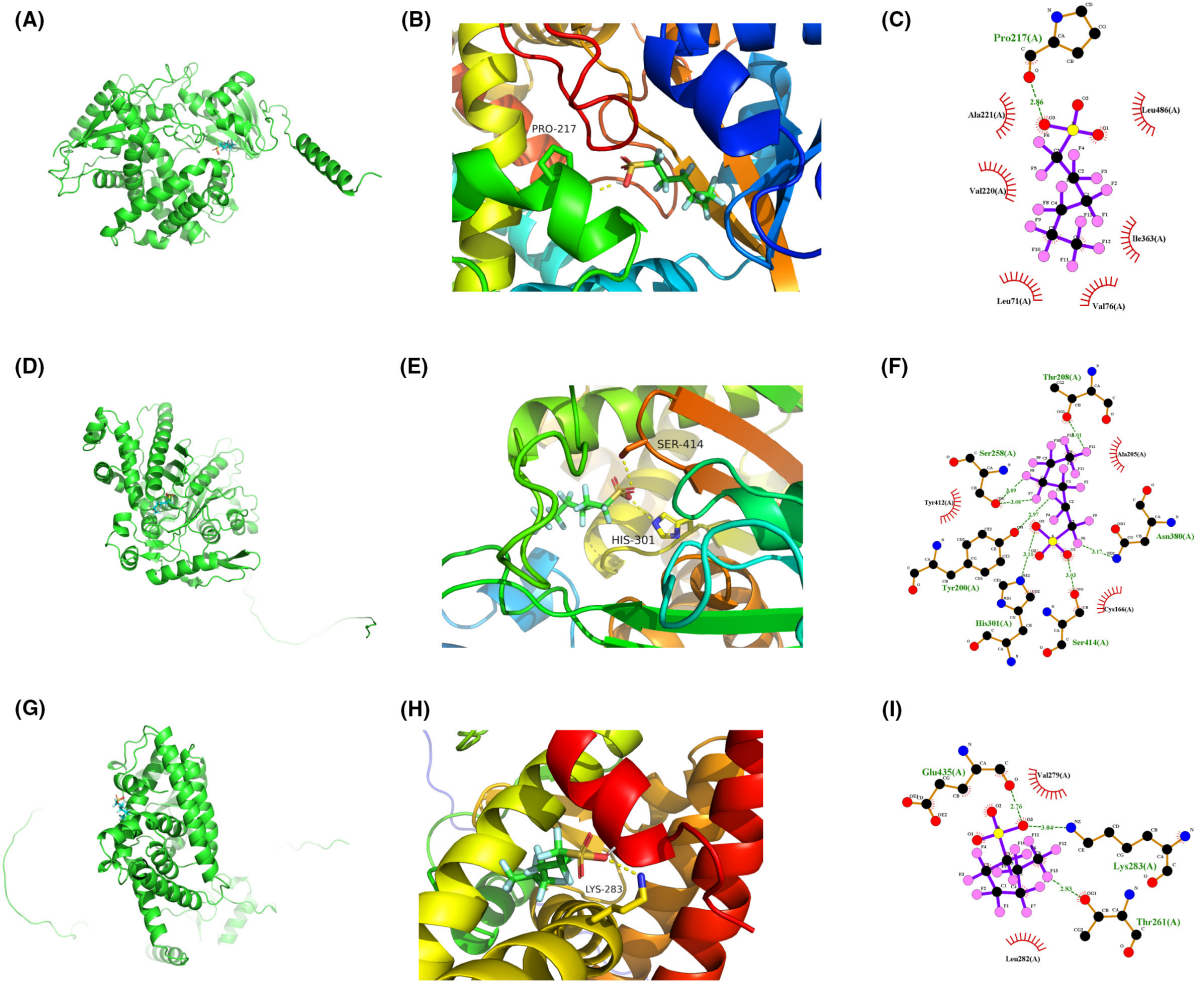
Molecular docking patterns for CYP7A1, HMGCS2, PPARD, and perfluorohexanesulfonic acid. (A–C) Schematic representation of the CYP7A1 protein structure (A), overall structure with docking results (B) and docking details revealing that the small molecule forms a 0.286 nm hydrogen bond with PRO‐217 in the protein, while VAL‐220, ILE‐363, and other residues contribute to hydrophobic interactions (C). (D–F) Representation of the HMGCS2 protein structure (D), overall structure with docking results (E) and docking details revealing that the small molecule forms hydrogen bonds with HIS‐301 and SER‐414 in the protein at 0.311 and 0.303 nm, respectively, and ALA‐205 and CYS‐166 contribute to hydrophobic interactions (F). (G–I). Schematic representation of the PPARD protein structure (G), overall structure with docking results (H), and docking details revealing that the small molecule forms a hydrogen bond with LYS‐283 at 0.304 nm, and LEU‐282 and VAL‐297 contribute to hydrophobic interactions (I).

**FIGURE 14:**
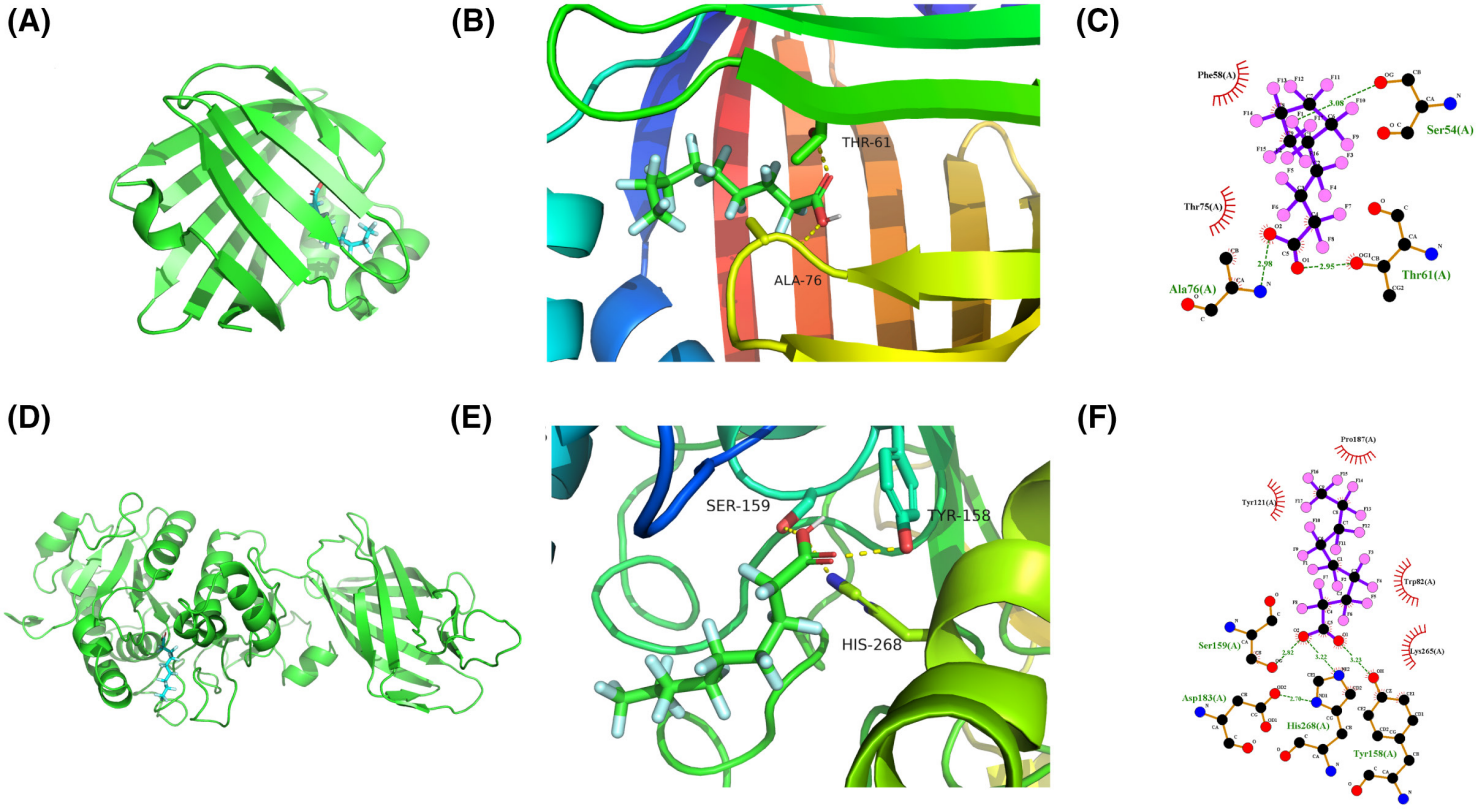
Molecular docking patterns for FABP4, LPL and triflumizole. (A–C) Schematic representation of the FABP4 protein structure (A), overall structure with docking results (B) and docking details revealing that the small molecule forms hydrogen bonds with THR‐61 and ALA‐76 at 0.295 and 0.298 nm respectively, and PHE‐58 and THR‐75 contribute to hydrophobic interactions (C). (D–F) Schematic representation of the LPL protein structure (D), overall structure with docking results (F) and docking details revealing that the small molecule forms hydrogen bonds with TYR‐158, SER‐159 and HIS‐268 at respective distances of 0.323, 0.282 and 0.322 nm, and TRP‐82 and LYS‐265 contribute to hydrophobic interactions (F).

**FIGURE 15:**
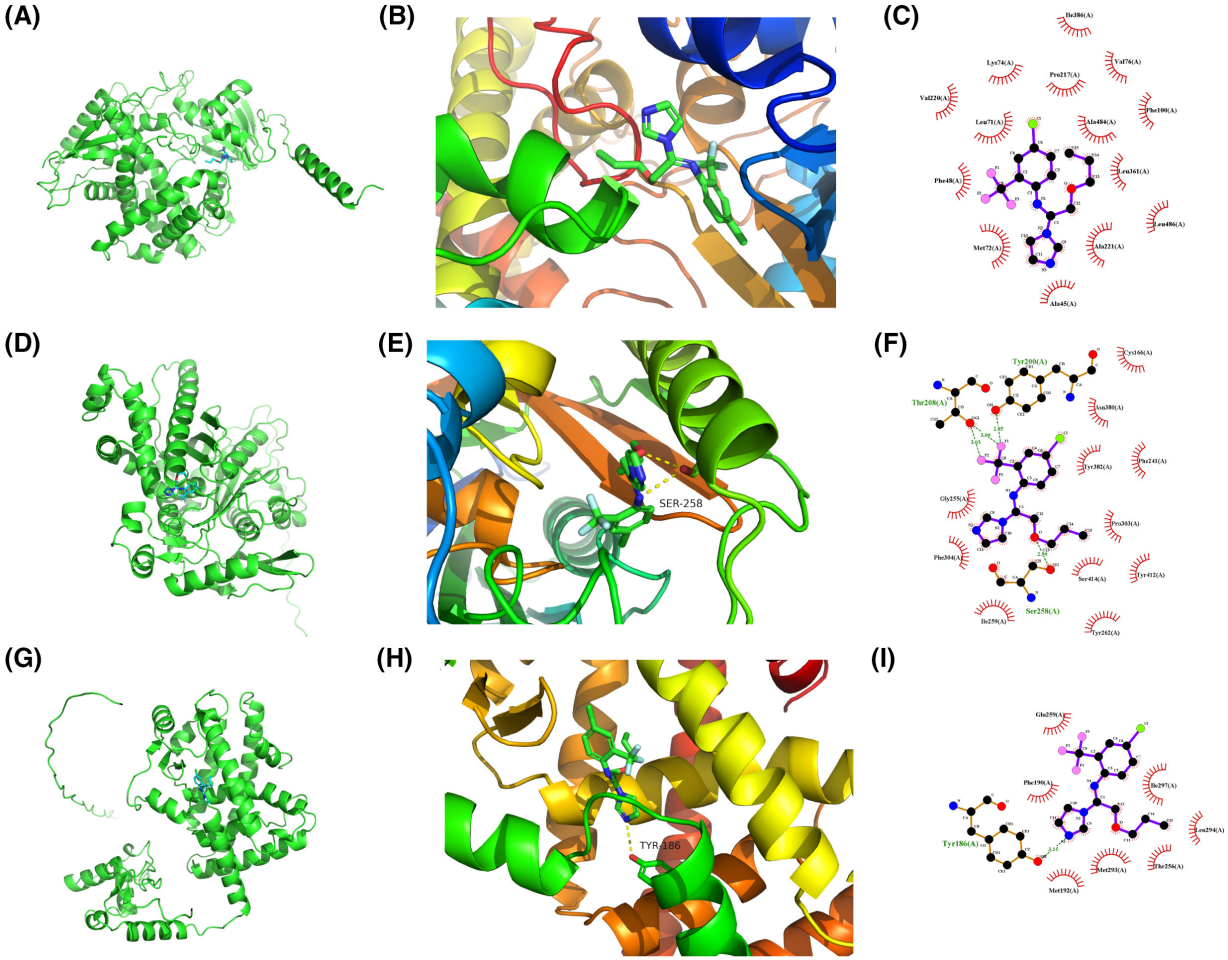
Molecular docking patterns for CYP7A1, HMGCS2, PPARD, and perfluorononanoic acid. (A–C) Schematic representation of the CYP7A1 protein structure (A), overall structure with docking results (B) and docking details revealing that VAL‐220 and MET‐72 surround the small‐molecule and the protein (C). (D–F) Schematic representation of the HMGCS2 protein structure (D), overall structure with docking results (E) and docking details revealing that the small molecule forms a hydrogen bond with SER‐258 at 0.299 nm, and GLY‐258 and ASN‐280 contribute to hydrophobic interactions (F). (G–I). Schematic representation of the PPARD protein structure (G), overall structure with docking results (H), and docking details revealing that the small molecule forms a hydrogen bond with TYR‐186 at 0.315 nm, and MET‐192 and PHE‐190 contribute to hydrophobic interactions (I).

The candidate drugs for the eight model genes were predicted using the enrichR database. The top 10 small molecule drugs and the corresponding genes are listed in Table 2. Perfluorohexanesulfonic acid, triflumizole, and perfluorononanoic acid were analysed further, and their molecular docking patterns with model genes are illustrated in Figures 13, 14, 15.

**TABLE 2:** Candidate drugs for model genes.

Name of drugs	*p*‐value	Adj *p*‐value	Combined score	Genes
Perfluorohexanesulfonic acid CTD 00004138	1.22E‐07	1.80E‐05	7945.654	HMGCS2; CYP7A1; PPARD
Triflumizole CTD 00002280	9.22E‐06	4.85E‐04	7722.366	FABP4; LPL
Perfluorononanoic acid CTD 00003375	1.53E‐07	1.80E‐05	7231.356	HMGCS2; CYP7A1; PPARD
Gemfibrozil CTD 00007055	2.28E‐07	1.80E‐05	6106.164	LPL; CYP7A1; PPARD
IBMX BOSS	3.24E‐07	2.19E‐05	5262.453	FABP4; LPL; PLIN1
Fenofibrate CTD 00006620	2.65E‐08	6.84E‐06	4038.463	FABP4; LPL; CYP7A1; PPARD
Bezafibrate CTD 00005506	2.89E‐08	6.84E‐06	3926.134	FABP4; LPL; CYP7A1; PPARD
25‐Hydroxycholesterol CTD 00000381	4.18E‐05	0.001364	2917.917	LPL; CYP7A1
Ethacrynic acid TTD 00007910	4.18E‐05	0.001364	2917.917	PLIN1; PPARD
Ethacrynic acid BOSS	5.26E‐05	0.001364	2521.841	PLIN1; PPARD

### 3.9 PPARD promotes the proliferation and migration of HCC cells

**FIGURE 16:**
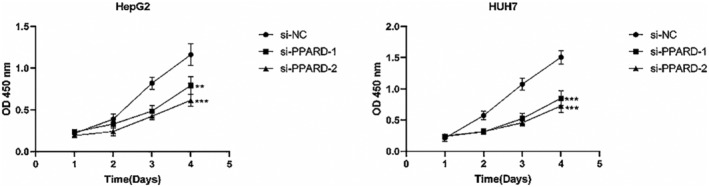
Graphs showing the proliferation rates of control and PPARD‐knockdown HepG2 and HUH7 cells after 48 h.

**FIGURE 17:**
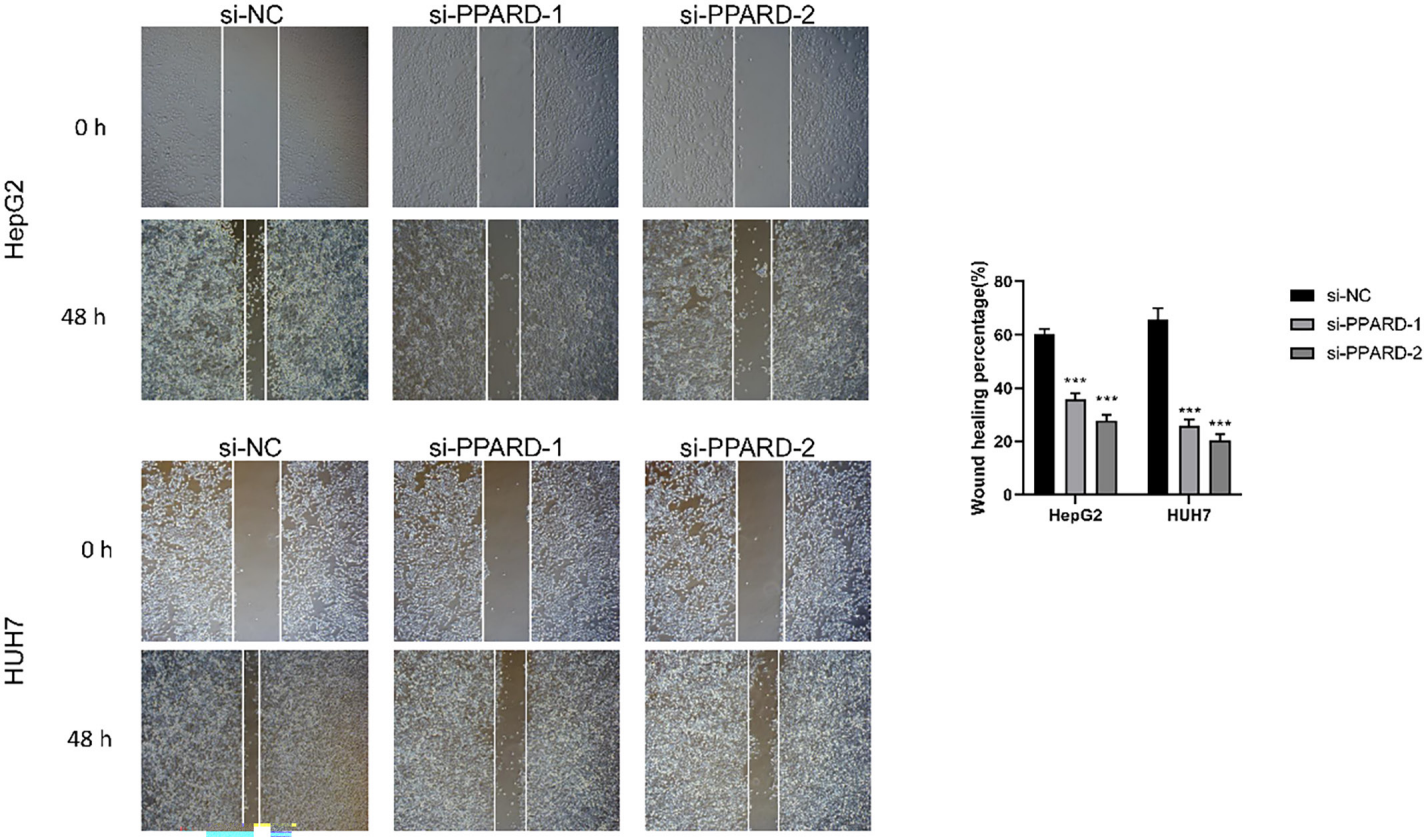
Representative images of the wound healing assay using control and PPARD‐knockdown HepG2 and HUH7 cells after 48 h.

To further ascertain the role of PPARD on the malignant potential of HCC cells, we knocked down the gene in the HepG2 and Huh7 cell lines using specific siRNA, and performed functional assays. The CCK8 assay revealed that the proliferation of liver cancer cells was significantly reduced after PPARD knockdown compared to that in the negative control group, and the results were consistent for both cell lines (*p* < 0.01; Figure 16). The migratory capacity of PPARD‐knockdown HepG2 cells was significantly lower compared to that of the control cells, as indicated by the smaller wound coverage area the former (*p* < 0.001); similar results were observed with the HUH7 cell line (Figure 17). Based on these results, we can surmise that PPARD promotes the proliferation and migration of liver cancer cells.

## 4 DISCUSSION

In spite of the advances in targeted therapy and immunotherapy, the OS rates of patients with advanced HCC remain suboptimal. Moreover, only a few patients benefit from targeted therapies and immunotherapy on account of tumour heterogeneity, thereby warranting novel therapeutic approaches.

PPARs are transcription factors involved in metabolism, inflammation, and cell differentiation, and are often dysregulated in metabolic disorders and cancers. PPARγ is the most extensively studied subtype, and its aberrantly high expression in HCC is associated with metabolic dysregulation and tumour progression.^32^ Moreover, since PPARγ overexpression plays a potential role in tumour immune escape, simultaneous inhibition of PPARγ and immune checkpoints can reshape the immune landscape of liver tumours, and overcome resistance to immune checkpoint blockers.^33^

We found that the hepatocytes had the most DEGs, as well as the highest PPAR score compared to the other annotated cell types in HCC. Furthermore, PPARα expression was also higher in the hepatocytes compared to other cells. PPARα levels decrease during alcoholic liver disease and non‐alcoholic steatohepatitis, which makes it a potential therapeutic biomarker for metabolism‐related liver diseases.^34^, ^35^, ^36^ Although PPARα‐mediated promotion or inhibition of liver tumorigenesis is still controversial, PPARα activation is certainly connected to HCC development.^37^, ^38^ In addition, the pathways enriched in the DEGs were mainly related to lipid and glucose metabolism, cell differentiation, and inflammation, all of which are regulated by PPARs. Thus, the PPAR‐related DEGs are possibly involved in HCC, although the underlying mechanisms need in‐depth exploration. Interestingly, the PPAR score was higher in normal hepatocytes than in tumour hepatocytes.

The PPARs are differentially expressed in hepatocytes, and the expression of PPAR subtypes depend on the hepatocyte subset.^39^ Nevertheless, the hepatocytes derived from normal liver and HCC samples had similar enriched hallmark pathways. This suggests that PPARs might regulate the same biological process in the normal and malignant although the expression patterns are distinct. Consistent with the lower PPAR score in the scRNA‐seq results, GSEA showed that the PPAR signalling pathway was significantly down‐regulated in tumour tissues. Therefore, we constructed a prognostic signature for HCC based on eight PPARs‐related genes, including FABP5, LPL, ACAA1, PPARD, FABP4, PLIN1, HMGCS2 and CYP7A1. These PPAR‐related genes also showed high frequency of somatic mutations in HCC patients, probably implying a positive response to immunotherapy. Given that the tumour mutational burden (TMB) is a predictor of immunotherapy response, higher somatic TMB is associated with better OS.^40^ Furthermore, the model genes are also established targets of PPARs.^41^ FABP5, LPL, ACAA1 and PPARD were identified as risk factors, and FABP4, PLIN1, HMGCS2 and CYP7A1 as protective factors. Since PPARD (PPARγ) was one of the model genes, we knocked down the gene in two HCC cell lines to further determine its biological role. Functional assays showed that PPARD is essential for the proliferation and migration of liver cancer cells.

To investigate the immune landscape of HCC, patients were classified into two clusters based on eight model genes by the ‘ConsensusClusterPlus’ package. Patients in Cluster 1 had a superior outcome compared to those in Cluster 2. Immune checkpoint genes, including YTHDF1, PDCD1, TNFRSF4/8/14, NRP1, LAG and JAK2, were highly expressed in Cluster 2. High expression of immune checkpoints in the tumour microenvironment is an important mechanism of immune escape, and blocking these molecules can augment the anti‐tumour immune response.^42^ In fact, increased expression of immune checkpoint genes in various cancers is associated with high response to immunotherapy and a favourable prognosis.^43^ Cluster 2 also exhibited greater infiltration of immune cells, including B cells, CAFs, CD8 T cells, cytotoxic lymphocytes, and so forth. The presence of tumour‐infiltrating immune cells is related to better clinical response and OS.^44^ Therefore, the Cluster 2 tumours can be described as immunologically ‘hot’, which translate to better response to immunotherapy, along with improved survival and quality of life.

Due to the critical role of PPARs in metabolic regulation, several PPAR agonists have been developed for the treatment of metabolic diseases, especially dyslipidemia and type 2 diabetes.^45^, ^46^, ^47^ To this end, we predicted candidate small molecule drugs for the eight PPARs‐related genes, and performed molecular docking analysis. The top three drugs were perfluorohexanesulfonic acid, triflumizole, and perfluorononanoic acid, none of which have been investigated in the context of HCC. The clinical feasibility of PPAR‐targeting small‐molecule compounds for HCC treatment warranty further exploration.

Currently, there is limited research on the establishment of PPAR‐related gene prognostic models. Zhang et al. confirmed that ACOX2 impedes the progression of HCC through the PPARα pathway using both bioinformatics and experimental approaches.^48^ However, they did not construct a prognostic model based on PPAR‐related genes, nor investigate the mechanisms of PPAR‐related genes in liver cancer. Our prognostic model was significantly associated with the survival status and pathological stage of HCC patients, and accurately predicted patient prognosis in the training set, as well as external validation sets (ICGI‐LIRI and GSE14520). Therefore, our prognostic model can prove to be a valuable tool to guide clinical decision‐making and improve outcomes of HCC patients. Overall, these findings indicate that PPAR‐related genes may influence the progression of liver cancer through complex regulatory networks, which is worthy of further investigation.

In conclusion, we established a prognostic model for HCC based on PPARs‐related genes. Our findings provide important insights into the development of personalized treatment strategies for patients with HCC. This novel prognostic model may prove to be a valuable tool for guiding clinical decision‐making and improving patient outcomes. Further validation studies and clinical trials are essential to ascertain the clinical utility of these findings and to evaluate the efficacy of potential immunotherapeutic targets identified in this study.

## AUTHOR CONTRIBUTIONS

**Yumeng Wang:** Data curation (supporting); formal analysis (supporting); writing – original draft (lead); writing – review and editing (equal). **Shuqiang Li:** Data curation (lead); formal analysis (lead); writing – review and editing (equal). **Zihang Liu:** Data curation (lead); formal analysis (lead); writing – review and editing (equal). **Xuanzheng Li:** Data curation (lead); formal analysis (lead); writing – review and editing (equal). **Yifan Yu:** Data curation (lead); formal analysis (lead); writing – review and editing (equal). **Hao Liu:** Conceptualization (lead); writing – original draft (supporting); writing – review and editing (equal).

## CONFLICT OF INTEREST STATEMENT

There is no conflict of interest among the authors.

## Supporting information


Data S1.

## Data Availability

The datasets can be downloaded from the TCGA (https://portal.gdc.com), GEO (http://www.ncbi.nlm.nih.gov/geo/), and ICGC (https://icgc.org/) websites.
