# Supplementary material for: Identification of PPAR‐related differentially expressed genes liver hepatocellular carcinoma and construction of a prognostic model based on data analysis and molecular docking
Source: J Cell Mol Med. 2024 Apr 23;28(8):e18304. doi: 10.1111/jcmm.18304 (PMC11037413; doi:10.1111/jcmm.18304)
Supplement: Supplementary file 1 — Data S1. [file JCMM-28-e18304-s001.docx]

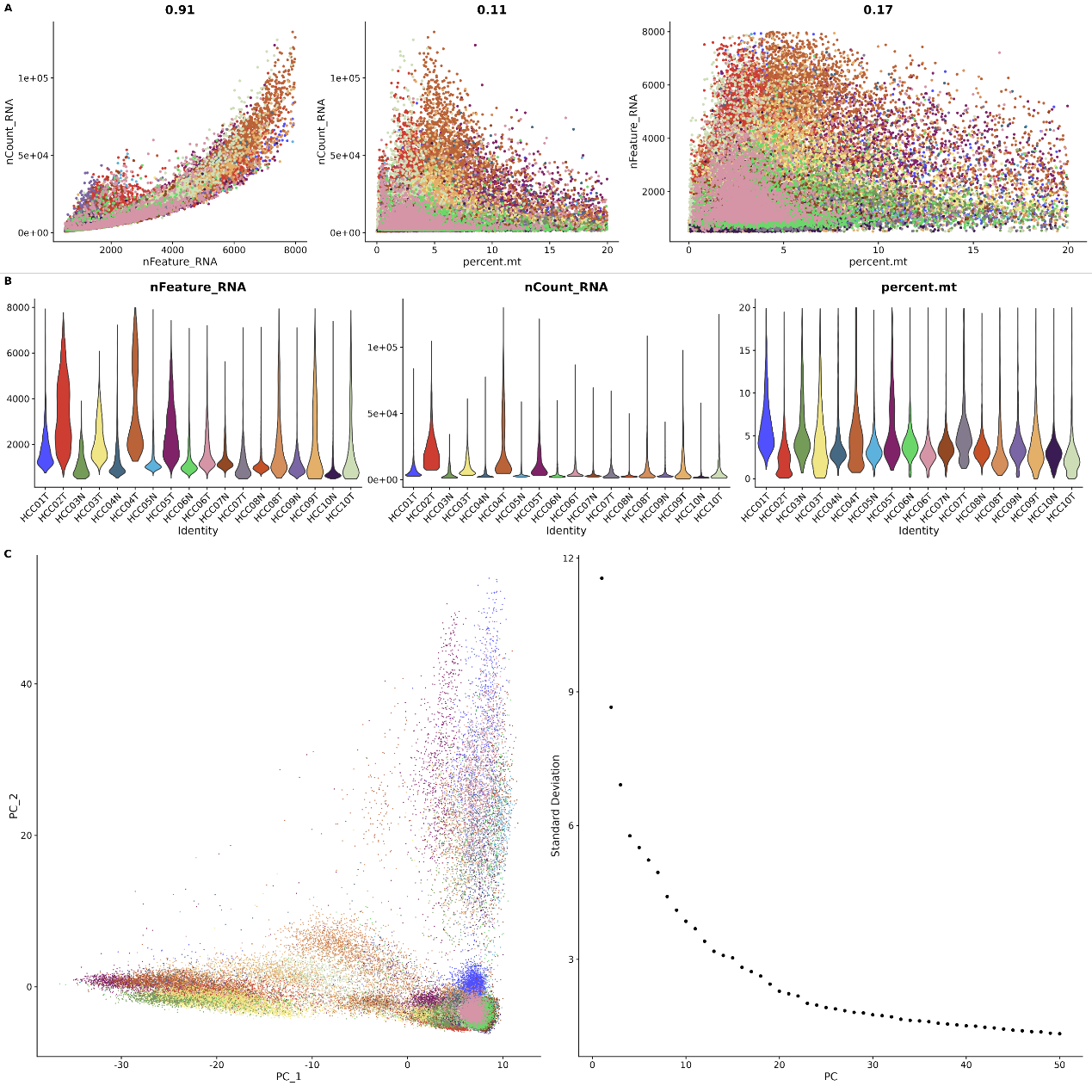


**Supplementary Figure 1. scRNA-seq analysis**. (A) it is important to examine the correlation between mitochondrial gene expression and the number of UMI/mRNA, as well as the relationship between the number of UMI and mRNA. (B) Quality control measures, such as assessing the number of unique genes and total molecules, and the percentage of reads that map to the mitochondrial genome, should also be included. (C) Principal component analysis (PCA) can be used to confirm the top 50 principal components based on scRNA-seq data.


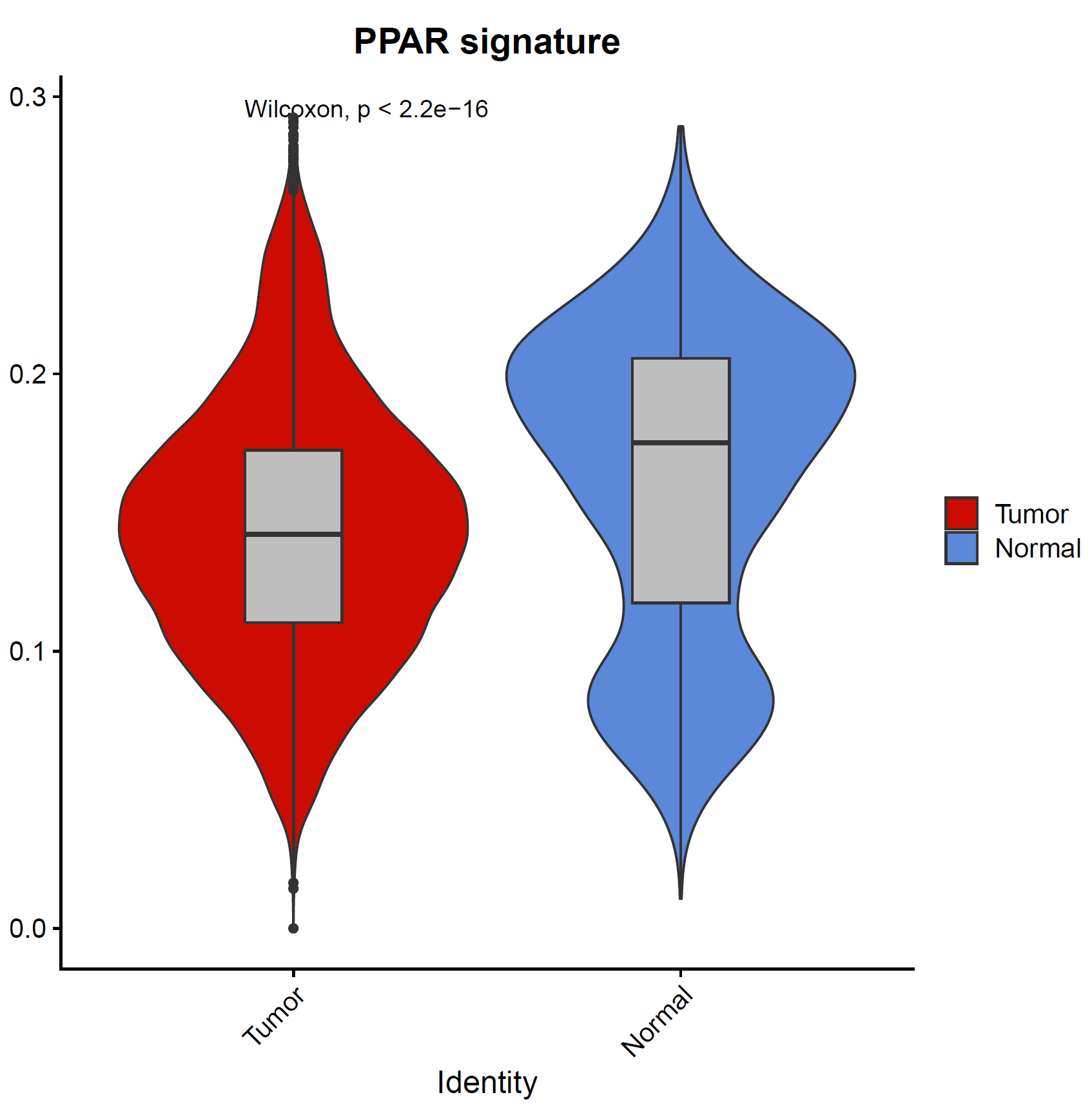


**Supplementary Figure 2.** Violin plot showing the PPAR signature score of hepatocytes between tumor and normal samples.


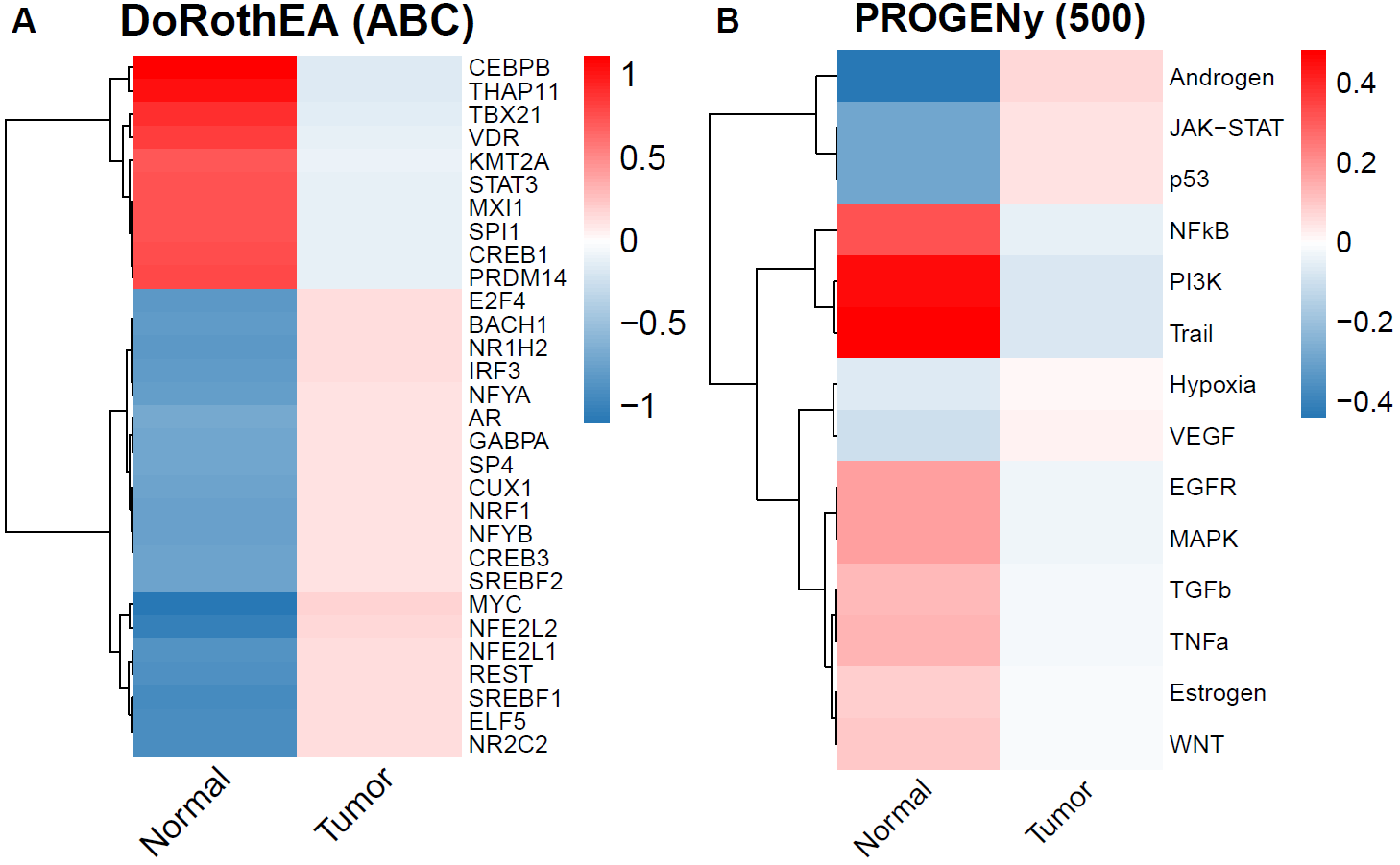


**Supplementary Figure 3. Transcription factor and tumor pathways signaling analysis.** (A) Heatmap showing the VIPER-inferred protein activity for the top 30 regulatory proteins in hepatocyte from normal and tumor samples. (B) Heatmap showing ProgenY scoring to annotate the tumor pathways in hepatocyte from normal and tumor samples.
